# Recurrence of methotrexate-induced leukoencephalopathy after methotrexate rechallenge: A case report and literature review

**DOI:** 10.1016/j.radcr.2022.11.057

**Published:** 2022-12-19

**Authors:** Ammar AlKawi, Amr Hanbali, Naser Haj Aissa, Mohammad Alaa Mufti, Saleha Abdul Rab

**Affiliations:** a Neuroscience Center, King Faisal Specialist Hospital & Research Center, Riyadh, Saudi Arabia; b Oncology Center, King Faisal Specialist Hospital & Research Center, Riyadh, Saudi Arabia; c College of Medicine, Alfaisal University, Takhassusi Rd, Riyadh 11533, Saudi Arabia

**Keywords:** Intrathecal methotrexate, Methotrexate-induced leukoencephalopathy, T-cell lymphoblastic leukemia, Neurotoxicity, Chemotherapy, Magnetic resonance imaging

## Abstract

Methotrexate (MTX) is potent chemotherapeutic agent, often administered intrathecally to treat or prevent central nervous system involvement in lymphomas and leukemias, particularly T-cell lymphoblastic leukemia (T-LBL). MTX has been linked to adverse neurologic effects that mimic acute stroke, including facial drooping, hemiplegia, impaired consciousness, and seizures, as well as changes on imaging—known as MTX-induced leukoencephalopathy (LE). We report a case of a 17-year-old male diagnosed with T-LBL, who had been receiving MTX chemotherapy for 4 months. After receiving his fourth dose of MTX, he presented to the emergency department with fever, facial drooping, and acute left-sided weakness. Brain magnetic resonance imaging (MRI) revealed bilateral deep white matter T2 hyperintense foci, increased on the right, with associated diffusion restriction in the right centrum semiovale—consistent with MTX-induced LE. After his symptoms resolved, he was discharged on leucovorin. Six months afterward, he was rechallenged with MTX and developed recurrence of symptoms. Repeat MRI showed well-defined T2/FLAIR hyperintensities in the right centrum semiovale without corresponding diffusion restriction. The left centrum semiovale hyperintensity became less conspicuous in comparison to the previous MRI study. We report a rare case of recurrence of LE after MTX rechallenge and discuss mechanisms, best imaging modalities, and possible treatment options for MTX-induced LE. Given the ominous presentation of MTX-induced LE, we urge clinicians to maintain a high index of suspicion for this condition. Further research is necessary to understand why only certain patients develop recurrence of LE after subsequent doses of MTX.

## Introduction

Methotrexate (MTX) is a competitive inhibitor of dihydrofolate reductase (DHFR) and therefore decreases the production of pyrimidine and purine nucleotides, making it a potent antimetabolite. It is used as a chemotherapeutic agent to inhibit the growth of cancer cells [1], and is indicated in the treatment of several neoplastic conditions such as leukemias and lymphomas, particularly T-cell lymphoblastic leukemia (T-LBL). Clinical administration of MTX may be referred to as high-dose MTX (HD-MTX) or low-dose MTX (LD-MTX). MTX is usually administered intravenously (IV) or orally, but given that acute leukemias and aggressive lymphoid malignancies often carry a high risk of central nervous system (CNS) involvement, it may also be administered intrathecally (IT) to prevent or treat CNS metastasis [2].

However, MTX has significant adverse effects, including neurotoxicity that may lead to severe neurologic morbidity, particularly with but not exclusive to IT administration. Leukoencephalopathy (LE) is the most common manifestation of MTX-induced neurotoxicity [3], and may present as focal neurological deficit in the form of headaches, confusion, or stroke, as well as seizures [4]. Severe cases may result in a progressive, destructive encephalopathy [3]. Due to its ambiguous presentation, acute MTX-induced LE poses a diagnostic and management challenge, particularly in the emergency department (ED). While the risk of developing LE increases after higher doses and more courses of MTX [5], it is uncommon for patients to develop LE again with subsequent doses of MTX (after developing it previously with resolution of symptoms). Herein, we report our experience in the diagnosis and management of a patient who developed acute MTX-induced LE, and was rechallenged with MTX 6 months later, where he developed LE once again. This case is reported in accordance with the CARE criteria [6].

## Case presentation

A 17-year-old male, a known case of T-LBL diagnosed 6 months earlier after the identification of a large mediastinal mass, presented to our ED with a fever of 38°C and left-sided weakness. Shortly afterwards while in the ED, he developed acute left facial droop, left arm weakness, and slurring of speech. Six months before his presentation to the ED, he had been started on induction chemotherapy (comprising of MTX) for the treatment of T-LBL. Four months after his diagnosis of T-LBL, he was started on interim maintenance therapy. Eight days prior to presentation to the ED, he had received his fourth dose of IV HD-MTX, followed by IT MTX given 24 hours later. Upon presentation, an acute ischemic stroke was suspected, and the patient was started on tissue plasminogen activator (tPA) and IV heparin. However, a computed tomography (CT) scan and computed tomography angiography (CTA) of the brain performed at the time revealed no evidence of acute insults, with no infracts or abnormalities.

**Fig. 1:**
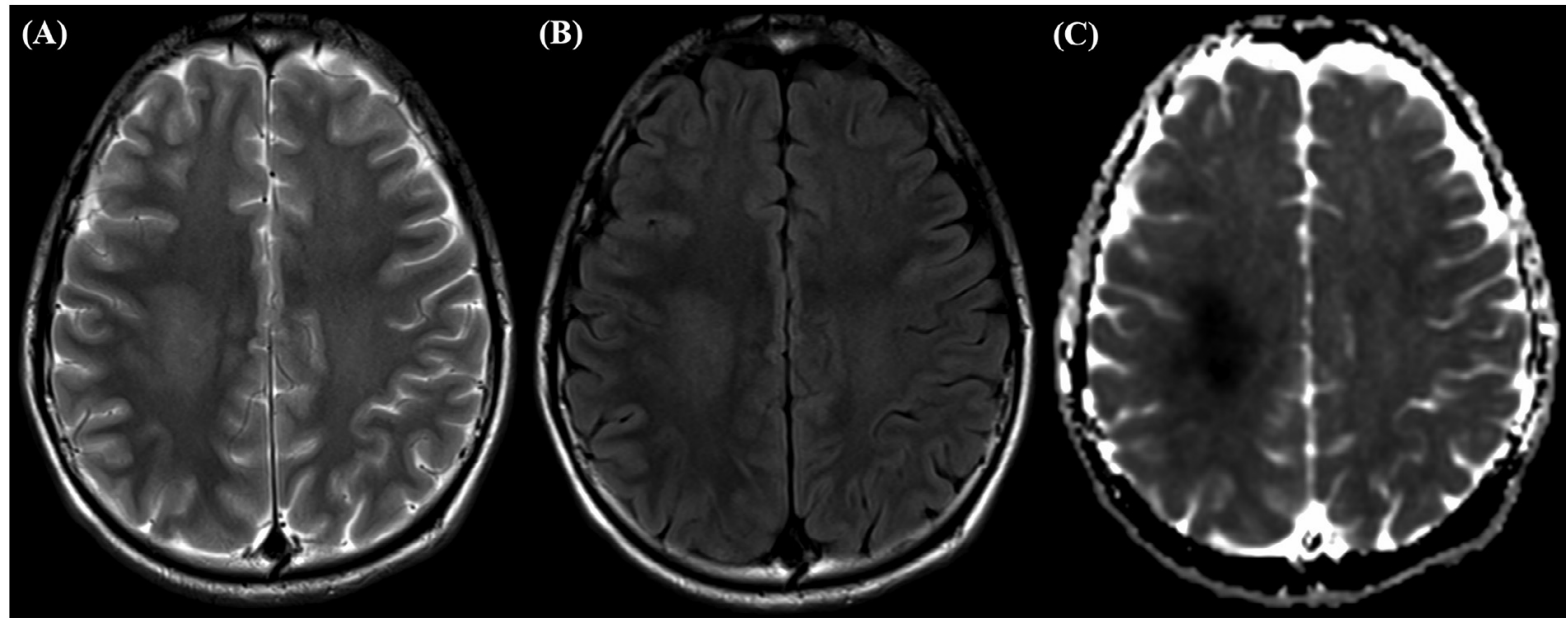
MRI findings taken at the time of presentation, revealing bilateral deep white matter hyperintense foci, greater on the right side, with associated diffusion restriction in the right centrum semiovale on (A) T2-weighted image, (B) FLAIR, (C) DWI.

Despite administration of IV thrombolysis, the patient continued to experience fluctuating weakness of the left upper and now lower extremities, raising doubts regarding the previous diagnosis of acute stroke, and brain magnetic resonance imaging (MRI) was recommended. The brain MRI revealed bilateral, deep white matter T2 hyperintense foci, greater on the right side, with associated diffusion restriction in the right centrum semiovale (Fig. 1), highly suggestive of MTX-induced LE. Leucovorin was administered, and a neurological assessment 6 days later revealed resolution of motor symptoms with no residual dysarthria. The patient was discharged, while continuing leucovorin. IV tPA administration caused no adverse effects.

**Fig. 2:**
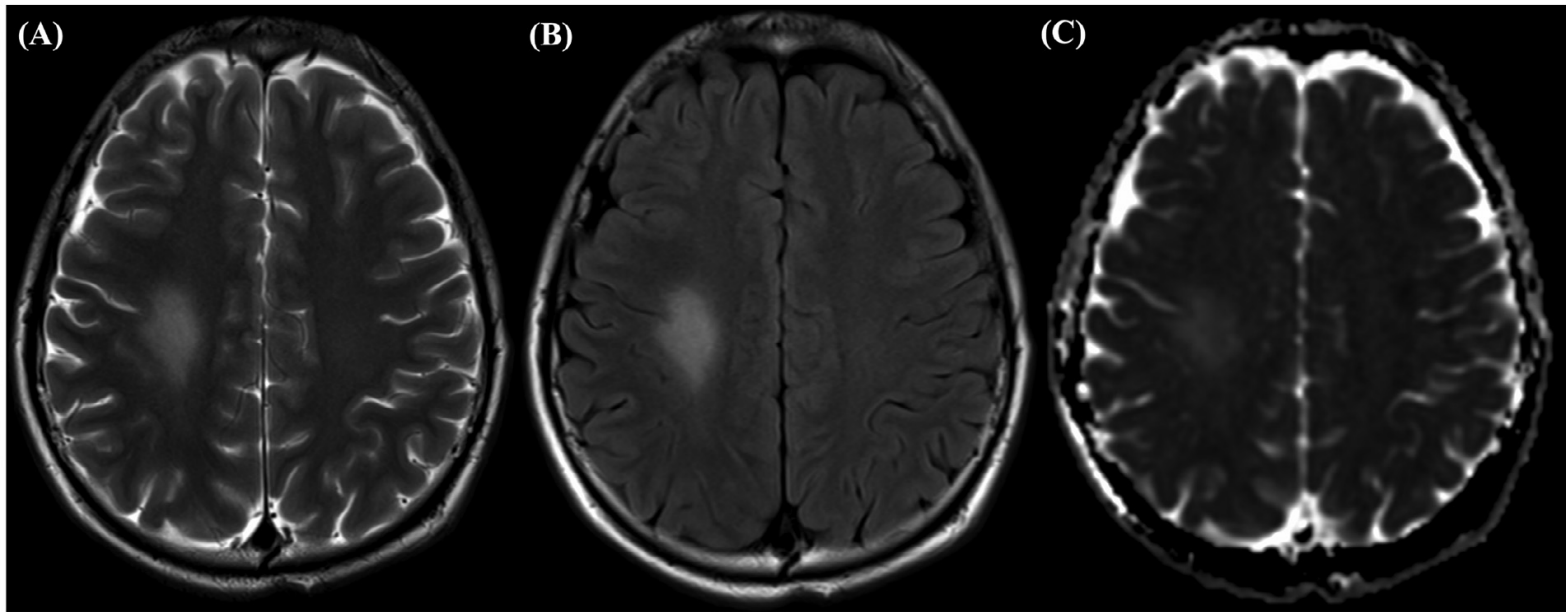
MRI findings taken one month after presentation, revealing asymmetric well-defined hyperintensity in the right centrum semiovale without corresponding diffusion restriction on (A) T2-weighted image, (B) FLAIR, (C) DWI.

Repeat MRI performed one month after discharge demonstrated asymmetric, well-defined T2/FLAIR hyperintensities in the right centrum semiovale without corresponding diffusion restriction (Fig. 2). The left centrum semiovale hyperintensity became less conspicuous in comparison to the previous MRI study.

**Fig. 3:**
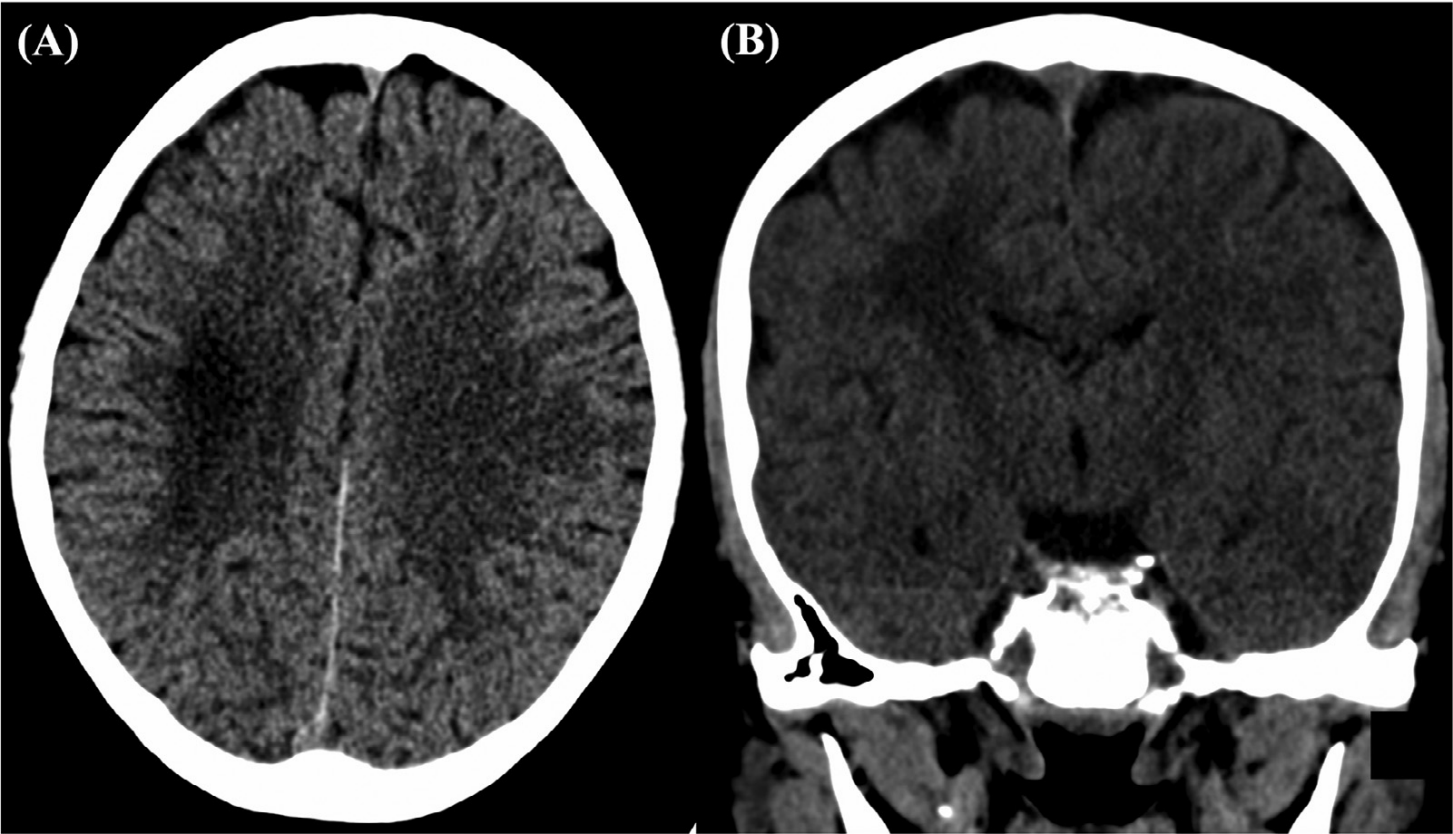
Re demonstration of a mass-like hypoattenuation within the posterior aspect of the right centrum semiovale, extending into subcortical white matter, consistent with known methotrexate-induced leukoencephalopathy.

**Fig. 4:**
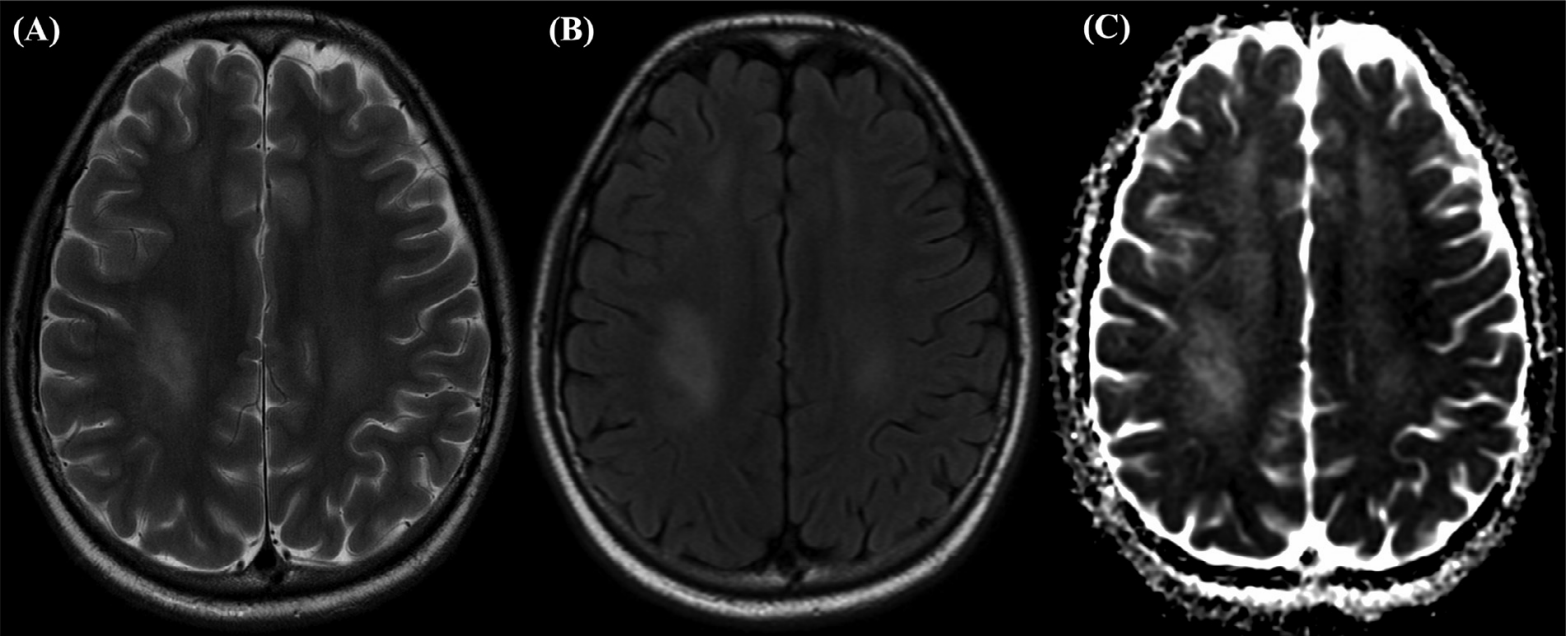
MRI findings taken 20 days after the second episode of MTX-induced LE, revealing further interval increase in the bilateral confluent high T2/FLAIR signal involving the corona radiata and centrum semiovale, however there is no diffusion restriction. These findings are suggestive of chronic leukoencephalopathy on (A) T2-weighted image (B) FLAIR (C) DWI.

Six months after his initial presentation to the ED, the patient was given 2 more doses of IV HD-MTX with a 5-day interval and presented 3 days later with acute right facial drooping, right-sided upper and lower limb weakness, dysarthria, and ataxia. A brain CT performed on arrival to the ER (Fig. 3) showed a residual mass-like lesion (an ill-defined hypoattenuation) in the right centrum semiovale, extending into subcortical white matter. This represented re-demonstration of the previous MTX-induced LE, again with no evidence of acute insults. Leucovorin was administered again, and his chemotherapy regimen was promptly changed. However, 20 days after the recurrence of MTX-induced LE, a brain MRI was performed (Fig. 4), revealing further interval increase in the bilateral confluent high T2/FLAIR signal involving the corona radiata and centrum semiovale when compared to the last MRI images taken (1 month after the first episode of LE). These images revealed no diffusion restriction but were indeed suggestive of chronic MTX-induced LE.

The last MRI images taken over a year after the first occurrence of LE revealed interval evolution of nonspecific FLAIR hyperintensities in the subcortical white matter/right centrum semiovale, with no signs of ongoing inflammatory changes—a sign that the patient's neurotoxicity was resolving. Currently, the patient (now 18 years old) continues to follow up with our outpatient clinic and is completing 7 cycles of maintenance chemotherapy for leukemia, with omission of MTX.

## Discussion

MTX is a folate analog antimetabolite that inhibits DHFR and consequently the formation of tetrahydrofolate. MTX is used as an antineoplastic drug or an immunosuppressant for immunoinflammatory diseases [7]. IT administration of MTX is sometimes preferred over IV or oral administration, for better reach into the CNS to eradicate CNS metastasis. Although MTX-related neurotoxicity is rare and occurs only in 3.1%-3.8% of cases [8,9], it manifests as alarming, acute-onset stroke-like symptoms, making prompt differentiation and diagnosis of the condition extremely critical. Symptoms include facial drooping, hemiplegia, disturbed consciousness, seizures, speech disorders, and unconsciousness [7,10]. Symptoms usually arise 5-13 days after IT or IV HD-MTX, followed by fluctuation for a few days and then complete resolution.

While the exact mechanisms causing MTX-induced LE are unclear, several have been suggested. Tetrahydrofolate is required for the synthesis of myelin and as MTX is a DHFR inhibitor, it may also inhibit myelin synthesis, possibly explaining the reversible LE [11]. Demyelination has also been attributed to decreased S-adenosylmethionine due to inhibition of DHFR, which normally helps in myelin sheath maintenance [12]. A third mechanism may be accumulation of adenosine in the cerebrospinal fluid (CSF), which interferes with neurotransmitter synthesis [13]. In a study by Bernini et al., CSF adenosine concentrations in patients receiving MTX, even in the absence of neurotoxicity, were greatly increased in comparison to controls, suggesting that MTX neurotoxicity may be mediated by adenosine [14].

The suggested treatments for MTX-induced LE include leucovorin (folinic acid), dextromethorphan, and aminophylline to help correct the metabolic derangement [15]. Notably, leucovorin serves as a source of tetrahydrofolate and quickly replenishes folic acid stores which would otherwise be inhibited by MTX [15], making it an effective rescue treatment. Combination treatment of the aforementioned drugs has also been proven to be effective [16].

In this article, we report our encounter with a 17-year-old patient diagnosed with T-LBL who presented with acute stroke-like symptoms 8 days after his fourth dose of IV and IT HD-MTX. Shortly afterwards, he was diagnosed with MTX-induced LE. Six days later, his symptoms had resolved, and he was discharged on leucovorin. Six months later, the patient presented again with acute stroke-like symptoms after being rechallenged with IV HD-MTX. The same findings seen on MRI after the first episode of LE were demonstrated once again, and the patient's chemotherapy was promptly changed. We present one of the few reports of a patient rechallenged with MTX who subsequently developed symptoms of LE again, which has rarely been seen in previous reports.

In an extensive study by Bhojwani et al., it was identified that only 1 of 13 patients rechallenged with intrathecal and/or high-dose MTX developed symptoms again. Patients received up to 20 doses of IT MTX without developing recurrence [9]. Other studies have also reported a zero-recurrence rate in nearly all patients rechallenged with MTX [17]. While guidelines on the matter are not yet clear, several authors have concluded that: (1) these incidents are benign, (2) that patients should be continued on MTX despite an episode of LE as it is an important chemotherapeutic drug, and (3) that patients can receive subsequent doses of MTX without recurrence of acute or subacute symptoms [8,9,17]. It is unclear why recurrence of symptoms occurred in our patient and is rarely seen in others. However, one genome-wide association study revealed that certain polymorphisms in genes responsible for neurodevelopmental pathways may play a role in MTX neurotoxicity, suggesting that a similar genetic cause may be responsible for the recurrence of symptoms in certain patients, and the absence of recurrence in nearly all others [9].

Furthermore, brain MRI findings in MTX-associated LE are vital to rule out stroke; however, presentation on imaging depends on whether the patient is symptomatic. In asymptomatic patients, these findings are best recognized on T2/FLAIR sequence as hyperintense lesions, while acutely symptomatic patients present with changes of atypical localizations, for example, in the supratentorial cortex, subcortical white matter, or thalamus [15]. Additionally, the T2 and/or FLAIR abnormalities usually persist after resolution of symptoms [7,9,18]. Our case presented very similarly to previous reports of MTX-induced LE following IT HD-MTX administration [15]; acute symptoms on presentation were associated with bilateral deep white matter hyperintense foci on T2, predominantly affecting the right hemisphere and associated diffusion restriction as shown in Fig. 1. However in our patient, a repeat MRI performed 1 month after the episode (Fig. 2) showed evidence of lingering LE despite leucovorin rescue—a finding not seen in previous reports of combination treatment with leucovorin [16]. Additionally, repeat MRIs performed 20 days after our patient's second incident of MTX-induced LE, showed signs of chronic LE once again, despite leucovorin administration. These findings, in combination with other studies that show persistence of MRI abnormalities after symptom resolution, suggest that perhaps leucovorin alone is not as efficient of a treatment for MTX-induced LE in the long-run as previously thought.

## Conclusion

MTX-induced LE is a significant adverse effect of IV and IT MTX administration and demands that clinicians be familiar with its presenting symptoms. Clinicians must maintain a high index of suspicion when handling patients on MTX chemotherapy who present with acute stroke-like symptoms, in order to correctly distinguish MTX neurotoxicity from clinical stroke. However, the approach to a child with MTX-induced LE is unclear, and whether subsequent doses of MTX should be administered after a single episode of LE or the regimen be changed is poorly understood. In this report, we present a case of a child who developed recurrence of stroke-like symptoms after being rechallenged with MTX—an occurrence that has rarely been seen previously. We also demonstrate a case where leucovorin administration did not result in complete resolution of the white matter T2 signal abnormality. Further research is necessary to understand why only certain patients develop recurrence of LE after subsequent doses of MTX while others do not. Lastly, we believe the utility of leucovorin as a rescue therapy for MTX-induced LE may need to be investigated further, to establish why leucovorin rescue does not resolve imaging abnormalities in all patients, while combination therapy appears to do so.

## Authors’ contributions

A.A. and A.H.—revision of the final manuscript, including medical writing for content, study concept or design. N.H.A., M.A.M., and S.A.R.—drafting of the manuscript for content, including medical writing for content; major role in the acquisition of data; study concept or design. All authors reviewed the manuscript for intellectual content and approved the submission.

## Ethics approval

The study was granted an exemption from requiring ethics pre-approval by the King Faisal Specialist Hospital & Research Center Research Ethics Committee as a case report with reference number 2215300.

## Patient consent

Written informed consent was obtained from the patient and their family for publication of this case report and accompanying images. Patient anonymity is maintained throughout this manuscript.

## Data availability statement

All data generated or analyzed during this study are included in this article and its supplementary material files. Further enquiries can be directed to the corresponding author.

## References

[1] Hannoodee M., Mittal M. (2021). StatPearls.

[2] Byrnes DM, Vargas F, Dermarkarian C, Kahn R, Kwon D, Hurley J (2019). Complications of intrathecal chemotherapy in adults: single-institution experience in 109 consecutive patients. J Oncol.

[3] Shuper A, Stark B, Kornreich L, Cohen IJ, Avrahami G, Yaniv I. (2002). Methotrexate-related neurotoxicity in the treatment of childhood acute lymphoblastic leukemia. Isr Med Assoc J.

[4] Salkade P., Lim T. (2012). Methotrexate-induced acute toxic leukoencephalopathy. J Cancer Res Ther.

[5] Reddick WE, Glass JO, Helton KJ, Langston JW, Xiong X, Wu S (2005). Prevalence of leukoencephalopathy in children treated for acute lymphoblastic leukemia with high-dose methotrexate. Am J Neuroradiol.

[6] Gagnier JJ, Kienle G, Altman DG, Moher D, Sox H, Riley D (2013). The CARE guidelines: consensus-based clinical case reporting guideline development. Glob Adv Health Med.

[7] (2018). Watanabe K, Arakawa Y, Oguma E, Uehara T, Yanagi M, Oyama C, et al. Characteristics of methotrexate-induced stroke-like neurotoxicity. Int J Hematol.

[8] Rubnitz JE, Relling MV, Harrison PL, Sandlund JT, Ribeiro RC, Rivera GK (1998). Transient encephalopathy following high-dose methotrexate treatment in childhood acute lymphoblastic leukemia. Leukemia.

[9] Bhojwani D, Sabin ND, Pei D, Yang JJ, Khan RB, Panetta JC (2014). Methotrexate-induced neurotoxicity and leukoencephalopathy in childhood acute lymphoblastic leukemia. J Clin Oncol.

[10] Rogers P, Pan WJ, Drachtman RA, Haines C (2017). A stroke mimic: methotrexate-induced neurotoxicity in the emergency department. J Emerg Med.

[11] Abromowitch M, Ochs J, Pui CH, Kalwinsky D, Rivera GK, Fairclough D (1988). High-dose methotrexate improves clinical outcome in children with acute lymphoblastic leukemia: St. Jude Total Therapy Study X. Med Pediatr Oncol.

[12] Surtees R., Clelland J., Hann I. (1998). Demyelination and single-carbon transfer pathway metabolites during the treatment of acute lymphoblastic leukemia: CSF studies. J Clin Oncol.

[13] Quinn C.T., Kamen B.A. (1996). A biochemical perspective of methotrexate neurotoxicity with insight on nonfolate rescue modalities. J Investig Med.

[14] Bernini JC, Fort DW, Griener JC, Kane BJ, Chappell WB, Kamen BA (1995). Aminophylline for methotrexate-induced neurotoxicity. Lancet.

[15] Cruz-Carreras MT, Chaftari P, Shamsnia A, Guha-Thakurta N, Gonzalez C.Cruz-Carreras M.T. (2017). Methotrexate-induced leukoencephalopathy presenting as stroke in the emergency department. Clin Case Rep.

[16] Jaksic W, Veljkovic D, Pozza C, Lewis I (2004). Methotrexate-induced leukoencephalopathy reversed by aminophylline and high-dose folinic acid. Acta Haematol.

[17] Rollins N, Winick N, Bash R, Booth T (2004). Acute methotrexate neurotoxicity: findings on diffusion-weighted imaging and correlation with clinical outcome. AJNR Am J Neuroradiol.

[18] Agarwal A, Vijay K, Thamburaj K, Ouyang T (2011). Transient leukoencephalopathy after intrathecal methotrexate mimicking stroke. Emerg Radiol.

